# Whole-Genome Resequencing Revealed Selective Signatures for Growth Traits in Hu and Gangba Sheep

**DOI:** 10.3390/genes15050551

**Published:** 2024-04-26

**Authors:** Peifu Yang, Mingyu Shang, Jingjing Bao, Tianyi Liu, Jinke Xiong, Jupeng Huang, Jinghua Sun, Li Zhang

**Affiliations:** Institute of Animal Science, Chinese Academy of Agricultural Sciences (CAAS), Beijing 100193, China; caasyangpeifu@163.com (P.Y.); shangmingyu263@sina.com (M.S.); baojingjing720@163.com (J.B.); liutianyi345@163.com (T.L.); jinkexiong2021@163.com (J.X.); h18766816604@163.com (J.H.); 82101222358@caas.cn (J.S.)

**Keywords:** resequencing, sheep, selection signals, growth traits

## Abstract

A genomic study was conducted to uncover the selection signatures in sheep that show extremely significant differences in growth traits under the same breed, age in months, nutrition level, and management practices. Hu sheep from Gansu Province and Gangba sheep from the Tibet Autonomous Region in China were selected. We collected whole-genome data from 40 sheep individuals (24 Hu sheep and 16 Gangba sheep), through whole-genome sequencing. Selection signals were analyzed using parameters such as F_ST_, π _ratio_, and Tajima’s D. We have identified several candidate genes that have undergone strong selection, particularly those associated with growth traits. Specifically, five growth-related genes were identified in both the Hu sheep group (*HDAC1*, *MYH7B*, *LCK*, *ACVR1*, *GNAI2*) and the Gangba sheep group (*RBBP8*, *ACSL3*, *FBXW11*, *PLAT*, *CRB1*). Additionally, in a genomic region strongly selected in both the Hu and Gangba sheep groups (Chr 22: 51,425,001-51,500,000), the growth-associated gene *CYP2E1* was identified, further highlighting the genetic factors influencing growth characteristics in these breeds. This study analyzes the genetic basis for significant differences in sheep phenotypes, identifies candidate genes related to sheep growth traits, lays the foundation for molecular genetic breeding in sheep, and accelerates the genetic improvement in livestock.

## 1. Introduction

Approximately 11,000 years ago, sheep were domesticated and later spread to various countries and regions around the world through human activities [1,2]. The earliest domesticated sheep provided meat products for humans. Around 5000 years ago, humans started to use sheep for secondary products, including skin, fur, and milk [3,4]. Recently, the surging market demand for mutton has made sheep breeding more and more focused on growth traits.

China leads the world in sheep farming volume. However, its market share in the international meat sheep market significantly trails behind that of developed countries, such as New Zealand and Australia. On the one hand, it is due to the high demand for lamb consumption in China, and on the other hand, it is also due to the weak competitiveness of lamb farming. According to statistics from National Bureau of Statistics of China (www.stats.gov.cn, accessed on 23 November 2023), the carcass weight of Chinese sheep in 2021 was only 15.5 kg, which is less than the world average (16.5 kg). The body weight of sheep reflects the production efficiency and breeding level of meat sheep. The improvement in the level of breeding is one of the most important ways to achieve efficient production and also one of the core competitiveness factors for the future development of the sheep industry. It is urgent to focus on growth traits and improve the yield performance of meat sheep through breeding techniques.

Sheep from different groups, isolated over time, undergo both natural and artificial selection. This long domestication process has led to distinct physical and appearance differences among groups. Through this selection, specific genes are strongly favored, resulting in phenotypic changes. These changes, known as selection signatures, have been a focus of recent research. Recent studies, including Gao [5] focused on Shanghai native pigs, Rafiepour [6] on water buffalos, and Rubin [7] on chickens, have utilized whole-genome resequencing to identify genes associated with growth traits. These findings offer valuable data for enhancing breeding programs and reducing the breeding cycle in livestock.

Using the ARS-UI-Ramb_v2.0 sheep reference genome and whole-genome resequencing data from 40 sheep, this study aims to identify genes that significantly affect sheep growth traits. These genes can be used as candidate genes for subsequent molecular verification tests, and the identified genes can be used to make chips and test the sheep population to obtain a high-quality sheep population. We aimed to provide a potential theoretical basis for advancing sheep breeding in China.

## 2. Materials and Methods

### 2.1. Ethics Statement

All animal experimental procedures were approved by the Ministry of Agriculture of the People’s Republic of China and the Institute of Animal Science, Chinese Academy of Agricultural Sciences and were performed according to the guidelines for the care and use of experimental animals established by this ministry. Ethical approval for animal survival was provided by the animal ethics committee of the Institute of Animal Science, Chinese Academy of Agricultural Sciences (IAS-CAAS) with the following reference number: IAS2019-61.

### 2.2. Animals and Sampling

In this study, sample collections of Hu sheep and Gangba sheep were conducted in Gansu Province and Tibet Autonomous Region, respectively. Both farms are intensive operations, implementing the same nutrition levels and management standards for pen-reared sheep of the same age group. Growth phenotype data of body weight, height, body length, chest circumference (measurement of the circumference at the thinnest part of the left front leg), and tube circumference were collected from all 6-month−old sheep that were not eaten in the morning in the farm, and we sorted the phenotypic values and selected 24 Hu sheep and 16 Gangba sheep (the same number of samples for the high and low phenotypes) within the range of 5% before and after. (As for the description of the phenotypes of Hu and Gangba breeds, see Section 3.1).

DNA was extracted from blood using a Wizard^®^ Genomic DNA Purification Kit (Promega., Madison, WI, USA). The extraction process entailed sample preparation with EDTA and Nuclei Lysis Solution. This was followed by tissue digestion using Proteinase K, and DNA purification through precipitation and centrifugation. Finally, the DNA pellet was rehydrated for storage. The NanoDrop 2000 spectrophotometer (Thermo Fisher Scientific Inc., Waltham, MA, USA) measured the concentration and purity of the DNA samples. For library construction and sequencing, we selected DNA samples with a concentration above 20 ng/µL and a purity ratio (A260/A280) of 1.8 to 2.0. Libraries for sequencing, derived from the manufacturer’s instructions (Illumina Inc., San Diego, CA, USA), were assembled according to the manufacturer’s guidelines and sequenced on the DNBSEQ−T7 platform using the PE150 model. The library preparation followed the NEBNext Ultra II DNA Library Prep Kit (New England Biolabs Inc., Ipswich, MA, USA) protocol for Illumina. This started with the sonication of DNA to a target size of 300 bp. Subsequent steps included end repair, A−tailing, and adapter ligation with NEBNext adapters. Size selection was performed using AMPure XP (Beckman Coulter Inc., Brea, CA, USA) beads to isolate the desired fragment sizes, followed by PCR amplification to enrich the library. The library’s quality and concentration were assessed using a Qubit 2.0 Fluorometer (Thermo Fisher Scientific Inc., Waltham, MA, USA) for quantification and an Agilent 2100 Bioanalyzer (Agilent Technologies Inc., Santa Clara, CA, USA) for the quality analysis.

### 2.3. Resequencing Data Processing, and SNP Calling

This analysis aims to index the sheep reference genome ARS−UI_Ramb_v2.0 and map the sequences of 40 samples to this reference using the Burrows Wheeler Aligner (Version: 0.7.17). First, establish an index of the sheep reference genome ARS−UI_Ramb_v2.0 using Burrows Wheeler Aligner. Then, map the opposite end sequences of 40 samples onto the reference genome. Next, convert SAM files to BAM files with Samtools (Version: 1.7) and sort the reads using the Picard toolkit. Duplicate reads are removed to ensure data quality. For SNP detection, the Genome Analysis Toolkit (Version: 4.3) was used. High−quality SNPs were identified with the following filters applied using VCFtools (Version: 0.1.15):

- Average sequencing depth > 3;
- Maximum deletion rate ≤ 0.9;
- Hardy–Weinberg equilibrium (HWE) < 10^−5^;
- Minor allele frequencies (MAFs) < 0.05.

Finally, filtered variant calls from all sample sets were used for imputation and phasing with Beagle (Version: 5.4), facilitating subsequent analyses.

### 2.4. Selective Scanning Detection

Based on the body weight data, 24 Hu sheep and 16 Gangba sheep were equally divided into high− and low−phenotype groups according to their weights. The genomes of the high− and low−phenotype sheep groups were compared to detect selective signals. This was achieved by scanning regions for nucleotide diversity (π), population differentiation value (F_ST_), and Tajima’s D using VCFtools (Version 0.1.15). Regions showing the top 5% of the statistical differentiation coefficient (F_ST_) and nucleotide diversity ratio (π _ratio_), and the lowest 5% of the Tajima’s D parameter, were identified as areas of interest. Candidate genes within these regions of interest were annotated using Bedtools for the further analysis.

### 2.5. Functional Enrichment Analysis

The enrichment analysis aims to identify functional clusters of candidate genes by using Kyoto Encyclopedia of Genes and Genomes (KEGG) pathways. Using DAVID (Version: 2023q4) (https://david.ncifcrf.gov/home.jsp, accessed on 3 February 2024) tools, perform the KEGG enrichment analysis. Determine the number of significant genes for each item using *p* < 0.05 as the significance threshold.

## 3. Results

### 3.1. Phenotype Value Data Statistics

**Figure 1:**
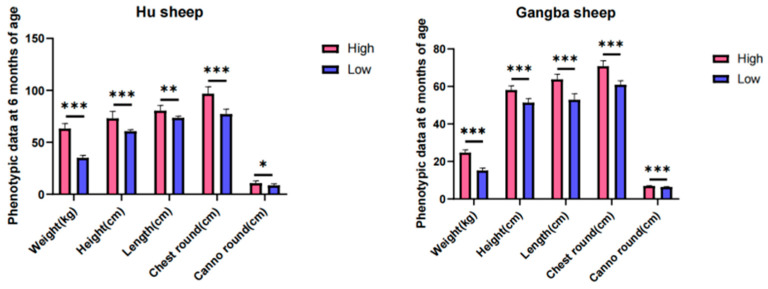
Comparative analysis of phenotypic values for growth traits in 6−month−old sheep classified by high− and low−growth states (Hu sheep on the **left**, Gangba sheep on the **right**). (In the figure, “*” indicates a significance level of *p* ≤ 0.05, “**” for *p* ≤ 0.01, and “***” for *p* ≤ 0.001.)

Typically, body weight is considered a critical metric for assessing growth status as it directly correlates with the individual’s overall dimensions and growth velocity. In conducting independent sample *t*−tests on the body weight of both high− and low−phenotype groups among Hu sheep, a *t* statistic of 17.83 and a *p*−value of 1.45 × 10^−14^ were recorded. For Gangba sheep, the high− and low−phenotype groups yielded a *t* statistic of 13.07 and a *p*−value of 7.43 × 10^−9^. Furthermore, the analysis extended to height, body length, chest circumference, and cannon bone circumference, with all *p*−values significantly below 0.01 (Figure 1). These data strongly support the existence of highly significant statistical differences between the high− and low−phenotype groups across the metrics.

### 3.2. Sequencing Data Statistics

**Figure 2:**
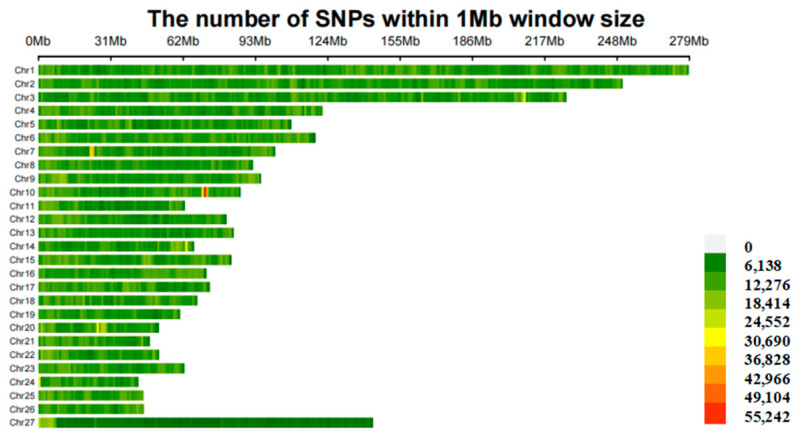
Density map of SNPs on the chromosomes.

We obtained whole−genome data from 40 sheep, including 24 Hu sheep (H) and 16 Gangba sheep (GB). The sequencing of the 40 sheep generated a total of 4.67 Tb of raw data, achieving an average depth of 42.35×, and the average depth consistency of the sample is high. We compared the sequencing data of the 40 sheep against the sheep reference genome ARS−UI_Ramb_v2.0, achieving an average alignment rate of 99.8%. After genotyping with GATK software(Version: 4.3.0.0) and a rigorous series of quality control and filtering steps, we identified a total of 26,500,815 SNPs (Figure 2).

### 3.3. Select Signal Analysis

**Figure 3:**
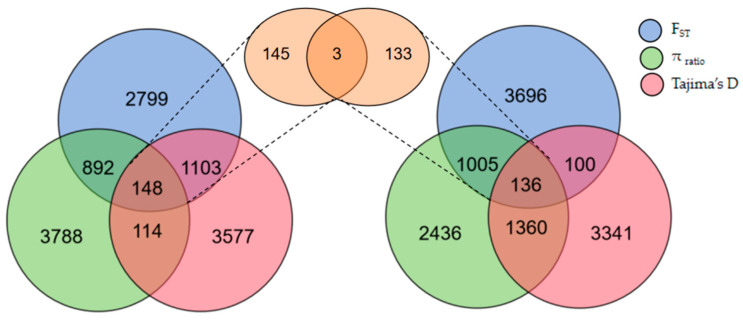
The number of genomic regions within the overlap window of the high− and low−phenotype groups of sheep (on the left is Hu sheep, and on the right is Gangba sheep) based on the 5% thresholds of F_ST_, π _ratio_, and Tajima’s D.

Based on their growth phenotypic values, 24 Hu sheep were divided into high− and low−phenotype groups. The sliding window method, with a 50 kb window and a 25 kb step, was used to scan for selection signals on the autosomes, utilizing a combination of F_ST_, π _ratio_, and Tajima’s D parameters. Selected genomic regions associated with the growth traits of Hu sheep were identified. An overlap region was pinpointed based on the top 5% threshold of F_ST_ and π _ratio_, along with the bottom 5% threshold of Tajima’s D value, indicating strong selection signals. Through selective scanning, a total of 148 genomic regions were screened (Figure 3). Annotating genes within the selected regions by Bedtools, 86 candidate genes that may be related to sheep growth were identified through gene annotation.

**Figure 4:**
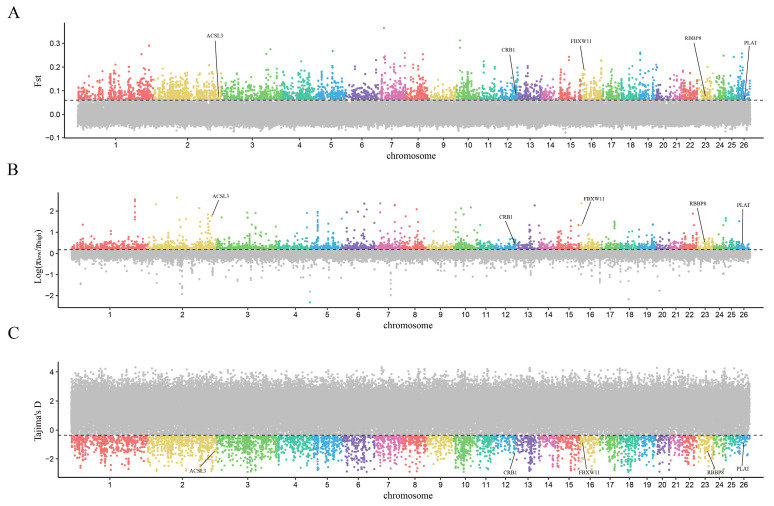
The genome−wide distribution of F_ST_ (**A**), π _ratio_ (**B**), and Tajima’s D (**C**) in Hu sheep (horizontal lines representing the top 5% of the entire genome). (Different colors represent different chromosomal regions, the gray part are regions not exceeding the threshold and therefore not of concern).

**Figure 5:**
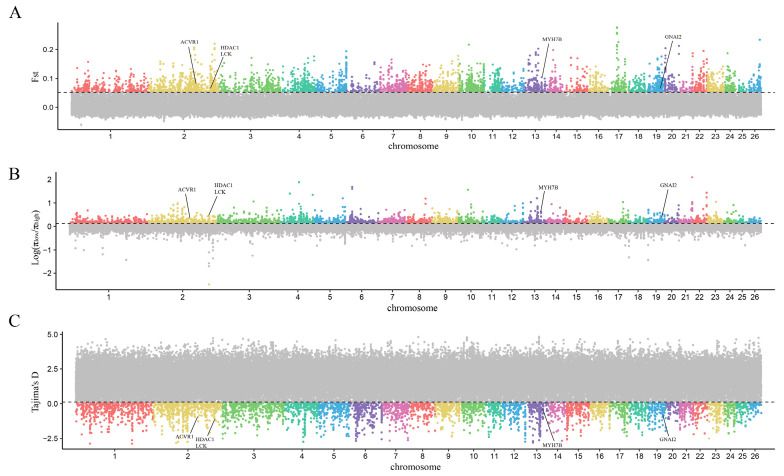
The genome−wide distribution of F_ST_ (**A**), π _ratio_ (**B**), and Tajima’s D (**C**) in Gangba sheep (horizontal lines representing the top 5% of the entire genome). (Different colors represent different chromosomal regions, the gray part are regions not exceeding the threshold and therefore not of concern).

Based on their growth status, 16 Gangba sheep were divided into groups of a high phenotype and low phenotype. Using the sliding window method, with a 50 kb window and a 25 kb step, we scanned the autosomes for selection signals, utilizing F_ST_, π _ratio_, and Tajima’s D parameters. The scanning identified 136 genomic regions and annotated 79 potential candidate genes related to sheep growth in the screened regions (Figure 3, Figure 4 and Figure 5).

Notably, through selective signal scanning, three overlapping contiguous genomic regions (Chr 22: 51425001−51450000, Chr 22: 51450001−51475000, Chr 22: 51475001−51500000) were obtained within 148 genomic regions of the Hu sheep group and 136 genomic regions of the Gangba sheep group. Annotations in three areas revealed two potential candidate genes associated with sheep growth: CYP2E1 and SYCE1.

### 3.4. Gene Enrichment and Analysis

**Figure 6:**
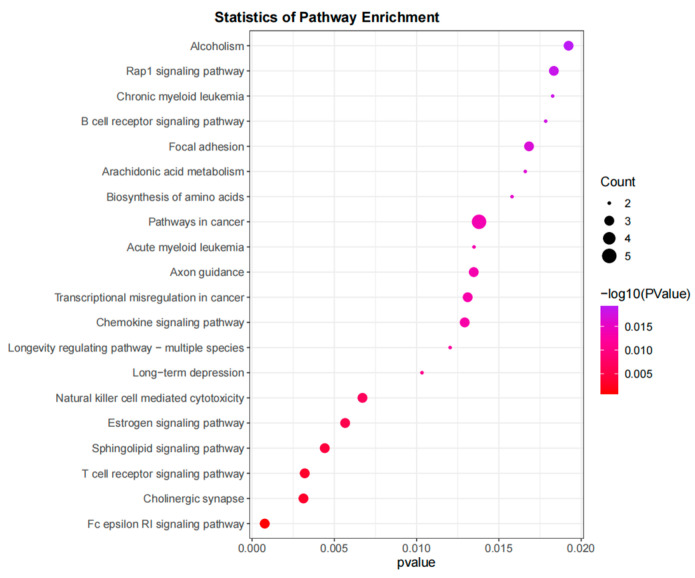
KEGG enrichment analysis (top 20) of selected genes in Hu sheep population.

The functional enrichment analysis conducted on the 86 candidate genes identified from the high− and low−phenotype groups of Hu sheep revealed that 40 KEGG pathways were found to be significantly enriched (*p* < 0.05), using DAVID, indicating a broad impact on various biological processes (Figure 6). These include cholinergic synapses, amino acid biosynthesis, arachidonic acid metabolism, and the synthesis, secretion, and action of parathyroid hormones, which are crucial for understanding the genetic basis of growth variations in Hu sheep.

**Figure 7:**
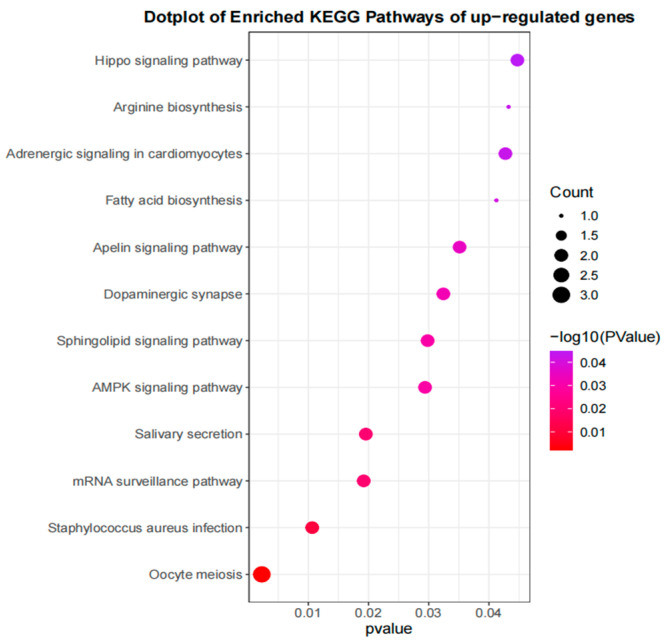
KEGG enrichment analysis of selected genes in Gangba sheep population.

The functional enrichment analysis of 79 candidate genes identified from the high− and low−phenotype groups of Gangba sheep revealed that 12 KEGG pathways were significantly enriched (*p* < 0.05), according to the criteria set for identifying key biological processes impacted by these genes (Figure 7). Pathways such as oocyte meiosis, AMPK signaling, and fatty acid biosynthesis were among those significantly enriched. These pathways are essential for understanding the molecular mechanisms underlying growth and development differences in Gangba sheep.

## 4. Discussion

### 4.1. Phenotypic Value Data and the Sequencing Data

In this study, the independent sample *t*−tests conducted between the high− and low−phenotype groups of Hu sheep and Gangba sheep showed very significant statistical differences (t statistic for Hu sheep was 17.83 with a *p*−value of 1.45 × 10^−14^; t statistic for Gangba sheep was 13.07 with a *p*−value of 7.43 × 10^−9^). These extremely low *p*−values indicate that the differences in body weight between the high− and low−phenotype groups of both sheep species are highly statistically significant, supporting the hypothesis that these weight differences may be driven by genetic factors. Further analyses of other phenotypes such as height, body length, chest circumference, and cannon bone circumference also found that all *p*−values were significantly below 0.01, further emphasizing the significant differences in multiple physical traits between different phenotype groups. These phenotypic data on body weight and dimensions are crucial indicators for assessing the growth status of sheep. Differences in body weight and other body dimensions can indicate the effects of specific genetic variations, which are critical for breeding program decisions.

The average sequencing depth of our 40 sheep samples reached 42.35×, indicating that the data have sufficient coverage for reliable genotyping and variant detection. High sequencing depth helps reduce the rates of false positives and false negatives in variant detection. The identification of 26,500,815 SNPs also indicates that the sheep genome has a rich genetic diversity. The discovery of these SNPs is also very important for finding genes and alleles that affect important agricultural traits.

### 4.2. Genes Associated with the Growth

In this study, we performed a comparative genomic analysis on populations of the same sheep breed that exhibit significant growth differences. Our aim was to identify the genes influencing sheep growth traits.

We identified five genes (*HDAC1*, *MYH7B*, *LCK*, *ACVR1*, *GNAI2*) associated with growth and development in Hu sheep in our study. Parallel studies in other species support these findings by highlighting the roles of these genes in various biological processes. In Medina’s study of dual−gene−knockout mice (*HDAC1* and *HDAC2*), it was found that the deprivation of *HDAC* 1/2 leads to senescence and loss of podocytes, impacting developmental processes [8,9]. In a mouse model, Zhou also found that knockdown of *HDAC1* promotes apoptosis of granule cells via the miR−202−3p−COX 1 axis, regulating sexual maturation [10]. Xu’s research indicates that *HDAC1* is expressed in the granulosa cells and oocyte nuclei of tan sheep, suggesting its critical role in follicle expression for the growth and maturation of oocytes [11]. *MYH7B*’s intron is known to regulate the expression of genes related to muscle growth, such as myostatin [12]. By influencing the development and density of placental villi, the *LCK* gene plays a critical role in regulating intrauterine development and birth weight [13]. *ACVR1*, a type I receptor for bone morphogenetic proteins (BMPs), which are essential regulators of chondrogenesis, significantly influences the proliferation and differentiation of chondrocytes [14]. Zhao’s comparative analysis of pigs, focusing on significant differences in fat and lean weight, demonstrated that *ACVR1* expression enhances myogenesis and increases muscle fibers [15]. The insulin−like growth factor (IGF) promotes growth, and its binding protein, *IGFBP3*, is among the most prevalent IGF−binding proteins in the bloodstream. Through qPCR, CCK8, and immunofluorescence analyses, Wang determined that *GNAI2*, targeted by *IGFBP3*, enhances the proliferation and suppresses the differentiation of Hu sheep skeletal muscle cells [16]. By knocking down *GNAI2* in mice, Huang discovered *GNAI2*’s role in regulating insulin sensitivity and glucose metabolism [17].

In Gangba sheep, five genes (*RBBP8*, *ACSL3*, *FBXW11*, *PLAT*, *CRB1*) were identified. Li identified six SNPs in close proximity to the *RBBP8* gene in broilers [18]. Using polyacrylamide gel electrophoresis, Lai detected three insertions and two deletions in the *ACSL3* gene’s intron. The subsequent analysis of growth trait associations revealed that polymorphisms in *ACSL3* significantly influence growth traits, including body weight [19]. Yuan studied the differential expression of *ACSL3* in the mature oocytes of adult and prepubertal pigs and concluded that the gene function may be related to oocytes [20]. The ubiquitin–proteasome system, which degrades various key regulatory proteins, is crucial for cellular homeostasis. *FBXW11* regulates cell proliferation by targeting proteins involved in the cell cycle or those degraded by this system, influencing animal development [21]. Schierding et al.’s GWAS analysis identified *FBXW11* as a crucial modifier of growth, functioning within the regulatory network [22]. Additionally, by sequencing the whole genome of two horses with notable differences in body size, Salek et al. identified the *CRB1* gene, examining selection signals using F_ST_ and π parameters [23]. Assessment of *PLAT* protein localization and expression in the immature cumulus–oocyte complex and at various stages of in vitro maturation revealed that *PLAT* postpones vesicular rupture in bovine oocytes by increasing cyclic 3′–5′ adenosine monophosphate levels during the initial 8 h of maturation [24].

It is noteworthy that three genomic regions overlapped between the Hu and Gamba sheep groups. The *CYP2E1* and *SYCE1* genes were annotated within the region, and the *CYP2E1* genes were identified to be associated with growth and development. *CYP2E1* is one of the primary cytochrome P450 mixed−function oxidases located in the liver and plays a pivotal role in the metabolism of xenobiotics [25]. Son conducted research on the livers of rats that had undergone hypophysectomy followed by glucose supplementation and found that *CYP2E1* is associated with the efficiency of glucose utilization [26]. Furthermore, studies by Wang revealed that the expression of the *CYP2E1* gene leads to increased oxidative stress and lipid peroxidation, which subsequently inhibits the growth of fetal rats [27].

This study utilized whole−genome resequencing data for selection signal scanning among sheep, comparing high− and low−growth groups to identify genomic differences associated with growth variations. The experiment conducted a population differentiation index (F_ST_), nucleotide diversity ratio (π _ratio_), and Tajima’s D analysis at the genome level, aiming to identify genes in significantly enriched KEGG pathways (*p* < 0.05). Our findings shed light on 148 potential selection regions encompassing 86 candidate genes within the high− and low−phenotype groups of Hu sheep. Notably, we pinpoint five key genes—*HDAC1*, *MYH7B*, *LCK*, *ACVR1*, *GNAI2*—linked to growth and development, thus corroborating and expanding upon previous genetic studies. Similarly, our analysis in Gangba sheep reveals five distinct genes—*RBBP8*, *ACSL3*, *FBXW11*, *PLAT*, *CRB1*—associated with growth phenotypes, further underscoring the utility of genomic approaches in elucidating trait−specific genetic variations. Furthermore, we identified three overlapping genomic regions between Hu and Gangba sheep groups, along with the annotation of the *CYP2E1* gene within these regions, offering new insights into sheep growth and development. And among the genes identified in our study, several (*MYH7B*, *LCK*, *RBBP8*, *PLAT*, *CRB1*, *CYP2E1*) have not been previously studied in sheep. The experimental validation of these genes is yet to be conducted. These findings not only augment our understanding of the genetic determinants of growth traits in sheep but also pave the way for targeted genetic improvements in breeding programs.

## 5. Conclusions

Of the 11 identified functional genes associated with growth traits, *CYP2E1* can be proposed as a key candidate gene for Hu sheep and Gangba sheep; *HDAC1*, *MYH7B*, *LCK*, *ACVR1*, and *GNAI2* as key candidate genes for Hu sheep; and *RBBP8*, *ACSL3*, *FBXW11*, *PLAT*, and *CRB1* for Gangba sheep. This study should accelerate genetic progress in future breeding work to increase the unit yield of meat sheep.

## Data Availability

The original contributions presented in the study are included in the article/Supplementary Materials, further inquiries can be directed to the corresponding author.

## Supplementary Materials

The following supporting information can be downloaded at: https://www.mdpi.com/article/10.3390/genes15050551/s1, Data S1. Sampling information of 50 sheep individuals in this study; Data S2. Details on putative regions experiencing selected sweeps (top 5% empirical cutoff) for FST values on each chromosome; Data S3. Details on putative regions experiencing selected sweeps (top 5% empirical cutoff) for LOG(π _ratio_) values on each chromosome; Data S4. Details on putative regions experiencing selected sweeps (bottom 5% empirical cutoff) for Tajimas’D values on each chromosome; Data S5. Functional annotation of the common regions based on the top 5% Fst and LOG (π _ratio_) values and bottom 5% Tajimas’D values; Data S6. Enrichment results of KEGG pathway (P value<0.05) for selected genes in 2 comparisons based on the top 5% Fst and LOG (π _ratio_) values and bottom 5% Tajimas’D values.
